# Evidence‐based framework for the management of disruptive physician behavior

**DOI:** 10.1002/jhrm.70010

**Published:** 2025-09-09

**Authors:** Allen M. Chen

**Affiliations:** ^1^ Department of Radiation Oncology University of California, Irvine, Chao Family Comprehensive Cancer Center Orange California USA

## Abstract

The consistent promotion of a culture of respect and accountability in the workplace is vital to the success of healthcare organizations. However, the existing literature on practical strategies for addressing misconduct, particularly with respect to physician behavior, is relatively sparse. The aim of this review was to thus devise an evidence‐based, empirical framework for the management and remediation of disruptive physician actions. Core themes on which to center the framework were initially identified based on the preferred reporting items for systematic review and meta‐analysis protocols (PRISMA‐P) statement. A MEDLINE search was undertaken to identify original peer‐reviewed works using terms associated with unprofessionalism with the goal of building a foundational basis. Articles published from January 2014 to March 2025 and restricted to the English language were included. Among the 1123 original articles that entered the final selection process, 1112 were excluded because they were focused solely on the characterization of disruptive behavior (*n* = 429); limited to trainees (*n* = 277), limited to ancillary staff (*n* = 150); concentrated on prevention (*n* = 148); and described consequences (*n* = 108). A total of 11 original publications thus met criteria for inclusion and differed in their design, methods, and endpoints. The core themes that emerged for framework construction were expectation setting (four studies); climate/organizational analysis (three studies); peer involvement (two studies); and professional training (two studies). The feasibility of developing an evidence‐based framework to address disruptive physician behavior was demonstrated. The management implications specific to risk are discussed.

## INTRODUCTION

The management of disruptive physician behavior has been an ongoing challenge for healthcare leaders. While the American Medical Association (AMA) has specifically defined disruptive behavior as “personal conduct—verbal or physical—that has the potential to negatively affect patient care or the ability to work with other members of the healthcare team, tangible, real‐world solutions for addressing such behavior are limited.^1^” This is practically relevant because the scope of this problem has been shown to be pervasive.^2^, ^3^ In one survey of nurses and physicians at more than 100 hospitals, a staggering 77% of respondents reported witnessing physicians engage in disruptive behavior, which was most typically classified as verbal abuse of another employee.^4^ Despite the agreement among stakeholders of the importance of addressing disruptive physician behavior, questions persist on the optimal manner to do so. To address this critical need, a comprehensive evidence‐based review was undertaken with the goal of constructing a practical framework to outline considerations which might be of utility in the management of disruptive physician behavior.

## METHODS AND MATERIALS

The design of this review originated from an internal grant that was awarded for the purpose of developing educational tools to raise awareness of professionalism issues, specifically as they relate to inclusion, civility and conduct in the setting of an expanding workforce. This systematic review was carried out following the Preferred Reporting Items for Systematic Reviews and Meta‐Analyses (PRISMA) guidelines with the objective of identifying peer‐reviewed publications reporting on strategies for the management of disruptive physicians. The initial screen was conducted on January 3, 2023, and repeated on a quarterly basis until March 2025.

To start, a MEDLINE literature search of publicly accessible publications was undertaken to identify original peer‐reviewed works pertaining to the topic of interventional strategies to address uncivility in the healthcare workplace. The search terms “professionalism,” “civility,” “conduct” “empathy,” “humanism” “inclusion,” “bullying,” “teamwork,” “conflict,” “disruptive,” “remediation,” and “communication” were broadly inputted in various permutations through the MEDLINE database to comprehensively initiate this exercise. To ensure that all possible publications were captured, multiple iterations of the search was processed. Boolean operators were routinely used to combine search terms, and advanced field tags were incorporated to refine the selection process in an attempt to limit the analysis to clinically oriented papers focused on healthcare. Reference lists from included articles were cross‐checked to identify additional articles. Review articles, narratives, editorials, commentaries, and papers presented as conference proceedings were excluded, as well as those originating from areas outside of the healthcare domain. Articles published from January 2013 to March 2025 with full text available and restricted to the English language and human subjects were included. The full bibliographies of identified articles were reviewed; and irrelevant studies and/or those of insufficient quality were selectively removed at the discretion of the investigator. Where individual works were included in multiple published series, the most complete or recent article was cited. To measure quality of the studies the National Institute of Health's quality‐measure tool for optimizing internal validity was used, and only those studies rated as “good” or “fair” were included.^5^

The evidence evaluating strategies for the remediation of disruptive behavior was evaluated using guidelines espoused by the JBI Manual for Evidence Synthesis focusing on Scoping Reviews. The PCC framework (population, concept, and context) was utilized as a guide to construct clear and meaningful objectives that could be used to construct the core framework for presentation.^6^ For the purpose of thematic development, basic qualitative content analysis using a descriptive approach to analysis and involving a process of open coding to allocate concepts or characteristics into overall categories was conducted.^7^  An interpretive synthesis of the available publications was then presented in both tabular and descriptive format.

## RESULTS

The initial search yielded 11,377 independent articles. After broad screening of these publications based on title and abstract, a total of 2970 studies proceeded to full‐text screening and were downloaded for review. Subsequently, 1847 articles were excluded because they were irrelevant to the topic of disruptive behavior (*N* = 952); review articles (*N* = 379); opinion pieces, letters, or editorials (*N* = 369); narratives (*N* = 74); conference proceedings or position papers (*N* = 41); or duplicative from prior works (*N* = 16). Another 16 publications were excluded because they originated from non‐medically related fields and/or were designed from a perspective outside of mainstream medicine.

Among the 1123 original peer‐reviewed articles that entered the final stage of the selection process and were reviewed in depth, a total of 1113 were then excluded for the following reasons: focused on the characterization of disruptive behavior with respect to prevalence, magnitude, environment, and/or severity without proposing any interventional strategies (*n* = 429); focused on disruptive behavior in medical students and/or physicians‐in‐training (*n* = 277); focused on disruptive behavior among nurses, medical assistants, and/or ancillary staff exclusively (*n* = 150); focused more on the identification of risk factors and/or determinants for disruptive behavior with more preventive than interventional intent (*n* = 148); focused on the consequences of disruptive behavior (*n* = 108).

**FIGURE 1:**
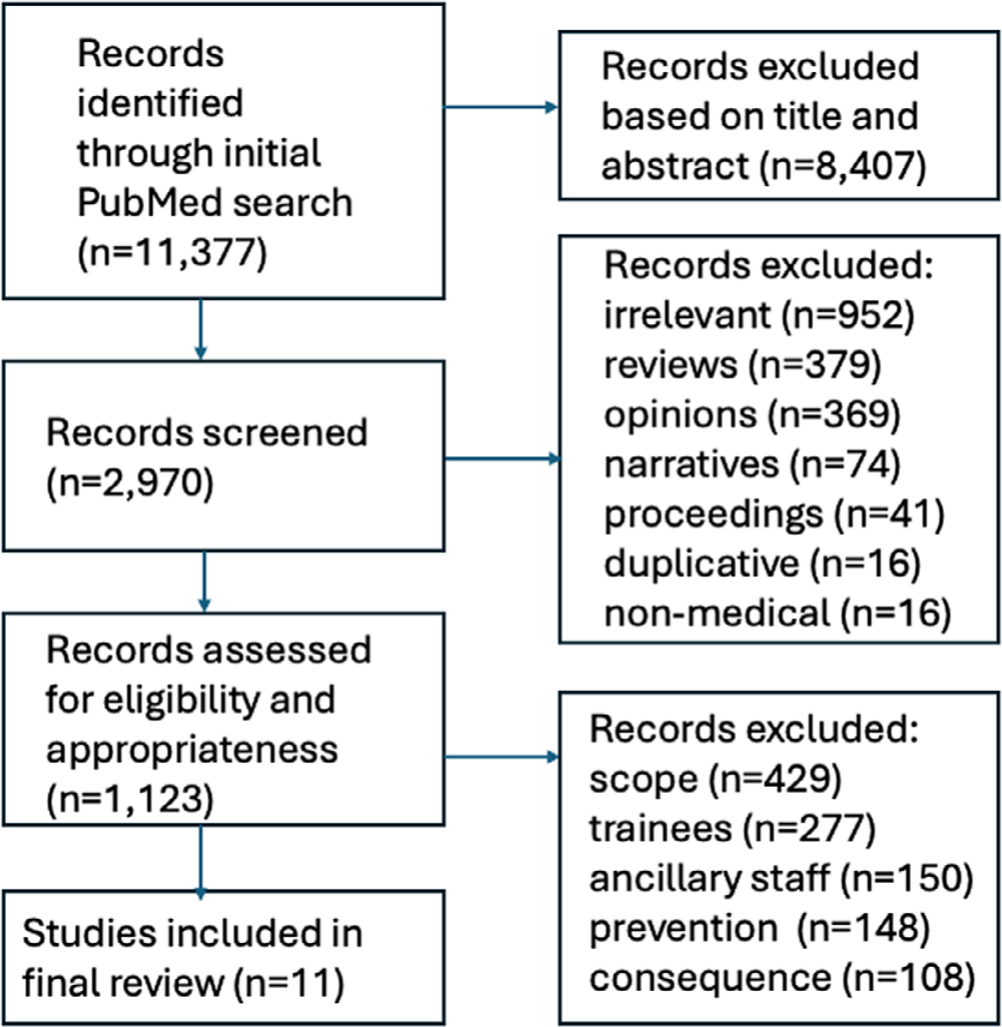
Schematic illustration of the search strategy.

**FIGURE 2:**
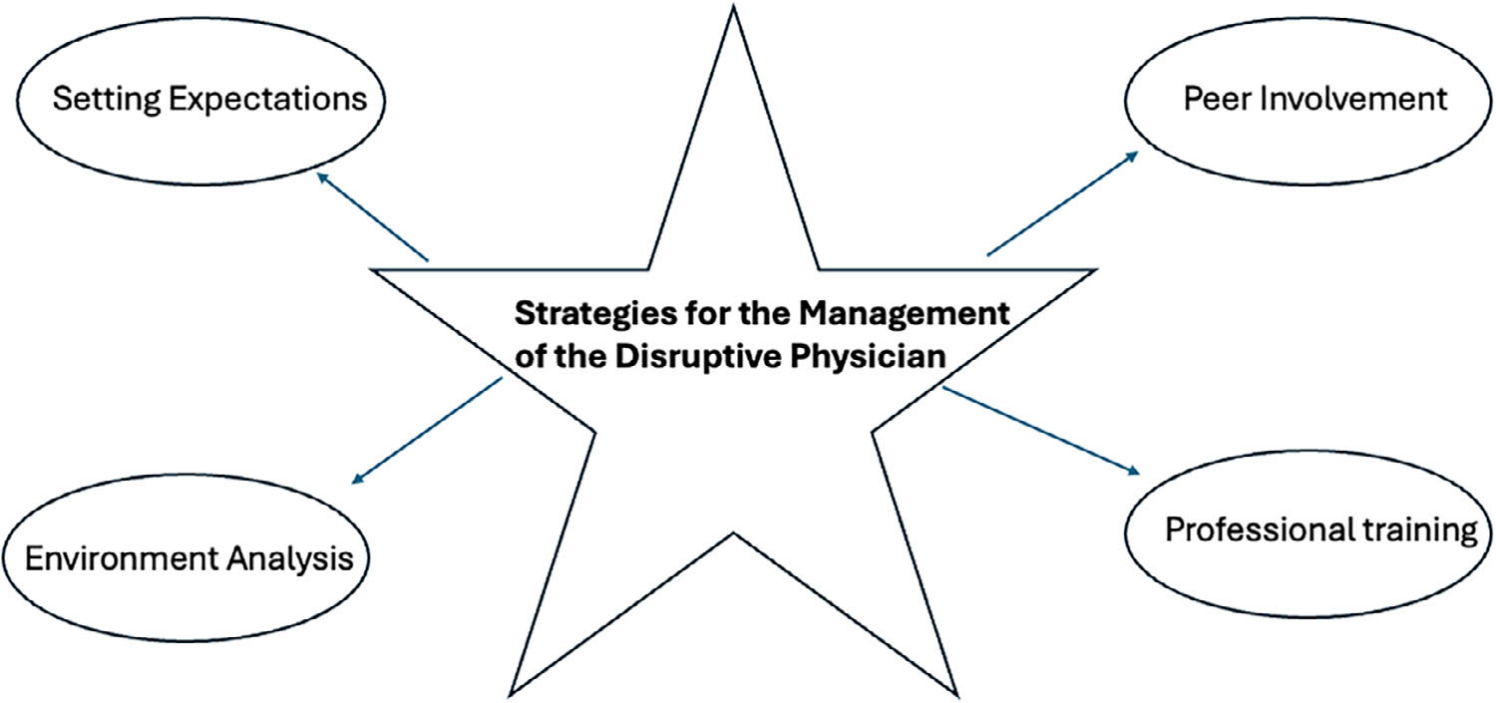
Themes that emerged from this evidence‐based review on the management of disruptive physician behavior.

A total of 11 original peer‐reviewed publications thus met eligibility criteria and formed the basis for this systematic review. A schematic illustration of the flowchart outlining the results of the search strategy is shown in Figure 1. All 11 publications identified were single‐institutional reports. Eight publications originated from academic medical centers; two from private industry; and one from a medical center affiliated with the armed forces. Nine were authored by investigators located in the United States, one was from Australia, and one was from Singapore. A total of three studies reported on longitudinal experiences involving prospective data collection. Table 1 outlines the details of the selected studies and provides a descriptive summary of key findings from the 10 peer‐reviewed publications that were identified. As shown in Figure 2, the dominant themes that emerged were setting expectations (four studies), climate/organizational analysis (three studies), peer involvement (two studies), and professionalism training (two studies).

**TABLE 1:** Summary of identified publications used in thematic construction.

Authors	Year	Intervention	Key findings	Theme
Webb et al.^27^	2016	CORS	Qualitative and quantitative data obtained over a 3‐year period involving 372 reports of disruptive behavior showed that co‐worker intervention and organizational guidelines limited the number of repeat offenders. Since the inception of CORS, 71% of those individuals who received a report of concern were not named in any subsequent reports during 1‐year follow‐up period after the behavior was brought to their attention	Peer involvement
Swiggart et al.^28^	2020	PDP	A total of 24 of 28 physicians who completed PDP (3 days followed by 3 daylong sessions spread over six months) which focused on systematic education in emotional intelligence, self‐awareness, introspection, interpersonal skills, conflict resolution, leadership, self‐care, emotional regulation, and mindfulness improved future behavior based on pre‐ and post‐course testing. Positive behaviors that increased after PDP included teamwork, peer relations, family balance, and empathy	Professional training
Penberthy et al.^29^	2018	ECCS	Longitudinal data collected among 46 participants from the ECCS course, which consisted of 3 initial consecutive days of training followed by three follow‐up 1‐day trainings at 1, 3, and 6 months for a total of up to 48 hours showed positive impact on system safety. Participants also reported improvements in quality of life, burnout, and emotional exhaustion	Professional training
Peisah et al.^30^	2023	Revalidation	The revalidation approach reinforced the values and principles of professional conduct and to establish institutional guidance that enabled individuals to reflect on their practice including lapses and achievements	Setting expectations
Ramirez et al.^31^	2025	Toxic Tree	This method emphasizes the examination of events from all perspectives, to promote wellness and communication, and to identify external or systemic causes	Climate analysis
Rosenstein^32^	2015	Progressive intervention	A system of gradual expectation setting from “coffee time” discussions to formal disciplinary action is described. Depending on the circumstances additional training in diversity management, anger management, stress management, or conflict management may be appropriate. More severe cases may require individualized coaching or counseling services	Setting expectations
Williams and Williams^33^	2020	EVLA	The EVLA framework attempts to address the underlying contributing factors and the environment in which the behavior occurs. The 5 components: Capacity, Capability, Readiness, Action, and Continuity. Interventions include assessment of health aspects that may contribute to misconduct and also the cognitive/emotional beliefs and values that provide the basis of behavior	Climate analysis
Finlayson et al.^34^	2013	FFDE	Data from 381 physicians referred for remediation showed that FFDE was effective at identifying physicians who might be unsafe to practice. Notably, physicians evaluated for disruptive behavior were found to be at significantly higher risk of being unfit than those referred for substance abuse, mental health, and/or sexual misconduct	Peer involvement
Hastie et al.^35^	2020	3E Model	3E model that focuses on cultural change through establishment of norms, education, and empowerment. What is emphasized is a collective effort with input from all organizational leaders. The norms of conduct are built by an organization, enforced by its leaders, and upheld by its members. Our learning and working environments should reflect those standards of professionalism, where deviations from behavior norms are not tolerated. Individuals should feel empowered to speak up and to come forward whether they are targets or bystanders of disruptive behavior, to protect their patients, their colleagues, and themselves	Climate analysis
Junga et al.^36^	2019	DEAL	4‐step “DEAL” model focused on determining the stakes, explaining intent, assessing consequences, and leveraging the future. This presents a general framework for on how to approach negotiations based on the 4 archetypes of the disruptive physician: the know‐it‐all, the insecure, the flake, and the combatant	Setting expectations
Lim et al.^37^	2021	3‐Prong Approach	Based on questionnaires sent to 1218 physicians and nurses, of which 500 replied, the authors developed a qualitative framework for addressing disruptive behavior based on respondent priorities. The 3‐prong approach focuses on (1) deterrence (feedback systems, reporting mechanisms), (2) development (training in communication, self‐awareness, emotional intelligence), and (3) demonstration (organizational leadership)	Setting expectations

Abbreviations: PDP, Program for Distressed Physicians; CORS, Co‐worker Observation Reporting System; ECCS, Effective Coping and Communication Skills; EVLA, Environmentally Valid Learning Approach; FFDE, Fitness for Duty Evaluation; DEAL, Determine the stakes, Explain the intent, Assess the consequences, Leverage the future.

## DISCUSSION

While the feasibility of developing a framework for approaching disruptive physician behavior in the healthcare workplace was demonstrated in this evidence‐based review, the findings from the present study pointedly illustrate the relative lack of literature on remediation strategies. It is particularly notable that while there is an abundance of publications describing the scope and breadth of unprofessional behavior, very few have reported on potential solutions.^8^, ^9^, ^10^, ^11^, ^12^, ^13^, ^14^, ^15^, ^16^, ^17^, ^18^, ^19^, ^20^ Given the inherently difficult nature of this subject, this observation was not particularly surprising. However, the implications are far‐reaching as they suggest that further research on evaluating the optimal methods in this setting are warranted.

The reasons underlying the lack of available literature deserve critical analysis. The are certainly multifaceted and likely relate to how discipline is viewed and practiced in the workplace. At the core is the lack of comfort among healthcare leaders in addressing misbehavior. Despite the fact that the Joint Commission expects hospitals to develop codes of conduct, define behavior norms, detail consequences for deviation from those norms, and “establish a process for managing disruptive behaviors,” it is apparent that persistent gaps in practice exist.^21^ Along these lines, in one survey of physician executives, only 17% of male and 11% of females strongly agreed that they were “well prepared to deal with disruptive behavior,” and 61% of respondents desired more training in confronting disruptive behavior.^22^

The perception of physician discipline as a low priority in healthcare might also be influenced by factors like burnout, administrative burdens, fear of litigation, and the emphasis on profit among systems. Of particular concern is what Zimmerman and Amori aptly term “the silent organizational pathology” of insidious intimidation, referring to a form of passive aggressive behavior in which confronting problems might be deterred because it is not convenient nor self‐preserving in intent.^23^ For instance, a poor culture as such might lead to the viewpoint of a double standard that permits high‐volume physicians (and powerful administrators) more leeway to engage in inappropriate conduct. Such perceived favoritism also may result in allegations of inappropriate inducements to profitable physicians and harsher treatment of their less‐profitable colleagues. Furthermore, evidence has suggested that physician discipline might not always be vigorously enforced, with some boards being more active than others in pursuing complaints.^24^ This can invariably lead to a sense that the system is not effectively holding physicians accountable for unprofessional conduct. All of these considerations point to a need to develop more standardized, evidence‐based methods to address disruptive behavior.

Of practical relevance is that disruptive physician behavior appears to be a continuing and widespread problem. In one survey from the American College of Physician Executives, greater than 70% of physicians noted that disruptive behavior occurred at least once a month in their organizations, and more than 10% indicated that the disruptive behavior occurred daily.^22^ As concerningly, it was also noted that 90% of the physicians surveyed believed disruptive physician behavior affected patient care.

It is important to recognize that disruptive behavior is a relatively nonspecific term that encompasses a litany of offenses, ranging in severity from minor to egregious.^25^, ^26^ The need to thoughtfully tailor remediation methods to the type of conduct is thus imperative. Given that discipline often carries a negative association with punishment and reprimand, which can deter research into more positive and constructive approaches, the emphasis on a learning culture is paramount.

Notably, the 11 studies that were identified in this systematic review varied significantly in size, design, and perspective.^27^, ^28^, ^29^, ^30^, ^31^, ^32^, ^33^, ^34^, ^35^, ^36^, ^37^ Most were small, retrospective experiences that were largely anecdotal and descriptive in nature, and all originated from a single institution. Nonetheless, some notable themes emerged across these studies which might be useful to develop a general framework for addressing disruptive physicians. The need to set clear expectations, to solicit organizational buy in, to invest in professional training, and to establish a collaborate process with peer involvement were all revealed.

The potential utility of peer involvement deserves particular mention. The Vanderbilt University experience with the co‐worker observation reporting system (CORS), a systems‐based platform for reporting of perceived disrespectful and unsafe conduct, demonstrated the importance of peer involvement in the intervention process.^27^ With CORS, selected peers meet with the potentially disrespectful individual, usually in an informal setting, to deliver feedback from a standardized assessment – then allows time for reflection. In their preliminary report, investigators showed that 71% of those individuals who received a report of some behavioral concern were not named in any subsequent reports during a 1‐year follow‐up period after the behavior was brought to their attention, and only 3% of the medical staff were associated with a pattern of CORS reports, meaning they received three or more CORS reports within a rolling 3‐year period.

The importance of professional development in the remediation process was also demonstrated. Swiggart et al presented prospective data on 28 physicians who had been previously cited for unprofessional behavior and who subsequently attended a program for distressed physician (PDP) course, an intensive 3‐day program (followed by 3 daylong sessions spread over 6 months) which focused on systematic education in emotional intelligence, self‐awareness, introspection, interpersonal skills, conflict resolution, leadership, self‐care, emotional regulation, and mindfulness.^28^ Using a specialized 35‐question survey—centered on personal demeanor, willingness or ability to keep up with hospital timeliness and tasks, bedside manner, and professional behavior—administered before and after the PDP, the investigators showed that 24 of the 28 physicians demonstrated a significant improvement in their understanding of conduct. This was powerful evidence that unprofessional behavior in physicians, as observed and reported by their peers and colleagues, can be positively modified by education. Pemberthy et al. similarly reported on an intensive 3‐day training program, Effective Coping and Communications Skills (ECCS) focused specifically on addressing unprofessional or distressed behaviors of physicians.^29^ Using data collected prior to the ECCS course and at 1‐, 3‐, and 6‐months after its completion, the authors showed significant improvement in how the 46 enrollees rated themselves with respect to burnout, quality of life, and emotional flooding scores.

Other investigators have focused on the role of the environment in helping to correct behavior. Peisah et al. outlined a Revalidation system, which was meant to reinforce the values and principles of professional conduct and to establish institutional guidance that enabled individuals to reflect on their practice including lapses and achievements.^30^ In a more recent study, Ramirez et al debated the contributions of the individual versus the environment (“bad apple or toxic tree”) in disruptive behavior and reaffirmed the importance of investing in community‐based education focused on wellness and communication to address offenders.^31^

The implications of disruptive behavior with respect to risk management are intuitive but warrant discussion.^38^ Without effective discipline and resultant remediation processes, organizations can suffer from low morale, decreased productivity, and increased turnover rates.  Furthermore, employees may become disengaged and dissatisfied if they perceive that poor performance or misconduct is not addressed consistently, leading to a toxic workplace atmosphere. Moreover, unresolved workplace conflicts can escalate, hindering teamwork and collaboration, and legal issues may arise if disciplinary policies are not enforced fairly, resulting in costly grievances or lawsuits.

Finally, the importance of preventive approaches, with respect to setting expectations, to disruptive behavior cannot be overstated.^39^, ^40^, ^41^ In a 2022 report, the Institute for Safe Medication Practices outlined recommendations for how organizations can pro‐actively promote workplace environments that deter disrespectful behaviors in healthcare.^42^ Among these included establishing a steering committee of employees from across the organization to create an action plan for addressing disrespectful behaviors; and creating a “no retribution” policy for people who report disrespectful behavior; cultural change to ensure systemic awareness, responsiveness and early intervention; staff education; policies and procedures that outline a consistent reporting and review process.

Given the relative lack of literature on the subject of how to address disruptive physician behavior, it is unclear what the most appropriate solutions might be in this setting. Opportunities for future research should thus focus on not only the development of new initiatives but also on the evaluation and comparison of methods using actionable endpoints. Data should be collected on the likelihood of sustained compliance to professional standards after any intervention, as well as on self‐rated knowledge of factors pertaining to awareness, communication, respect, and emotional intelligence. The need to assess the impact on systems‐based measures related to culture, patient safety, and risk management should also be prioritized.

## CONCLUSION

Despite the increased recognition of the prevalence of disruptive behavior in the healthcare workplace and its widespread consequences, solutions for addressing misconduct are lacking. The development and evaluation of effective remediation strategies are thus of critical importance to optimize organizational performance. While the strategies that were uncovered in the present study offer a practical means of addressing disruptive behavior which could aid healthcare leaders in addressing disruption, considerable improvement is needed to guide decision‐making in the future.

## CONFLICT OF INTEREST STATEMENT

The author declares no conflicts of interest.

## FUNDING INFORMATION

This work was funded by the University of California, Irvine, M‐POWER program.

## Data Availability

There is no original data arising from this work.

## References

[1] Physicians with disruptive behavior. Code of Medical Ethics of the American Medical Association: Current Opinions with Annotations. American Medical Association, Chicago, 2015 351‐353. https://code‐medical‐ethics.ama‐assn.org/ethics‐opinions/physicians‐disruptive‐behavior

[2] Leape LL and Fromson JA . Problem doctors: is there a system‐level solution? Ann Intern Med. 2006; 144:107‐115. 16418410 10.7326/0003-4819-144-2-200601170-00008

[3] Fujimoto M , Shimamura M , Miyazaki H . Bulwark effect of response in a causal model of disruptive clinician behavior: a quantitative analysis of the prevalence and impact in Japanese general hospitals. Healthcare. 2025; 13: 510. 40077072 10.3390/healthcare13050510 PMC11899433

[4] Rosenstein AH and O'Daniel M . A survey of the impact of disruptive behaviors and communication defects on patient safety. J Comm J Qual Patient Saf. 2008; 34: 464–471. 10.1016/s1553-7250(08)34058-6 18714748

[5] National Institute of Health, Study Quality Assessment Tools , https://www.nhlbi.nih.gov/health‐topics/study‐quality‐assessment‐tools

[6] Aromataris E , Lockwood C , Porritt K , Pilla B , Jordan Z , eds. JBI Manual for Evidence Synthesis. JBI; 2024.

[7] Pollock D , Michah DP , Khalil H , et al. Recommendations for the extraction, analysis, and presentation of results in scoping reviews. JBI Evid Synth. 2023; 21: 520–532. 36081365 10.11124/JBIES-22-00123

[8] Villafranca A , Hiebert B , Hamlin C , et al. Prevalence and predictors of exposure to disruptive behaviour in the operating room. Can J Anaesth. 2019; 66:781–794. 31168769 10.1007/s12630-019-01333-8

[9] Villafranca A , Hamlin C , Enns S , Jacobsohn E . Disruptive behaviour in the perioperative setting: a contemporary review. Can J Anaesth. 2017; 64:128–140. 27900669 10.1007/s12630-016-0784-x PMC5222921

[10] Kimes A , Davis L , Medlock A , et al. ‘I'm not calling him!’: disruptive physician behavior in the acute care setting. Medsurg Nurs. 2015; 24: 223–227. 26434034

[11] Hopkins J , Hedlin H , Weinacker A , et al. Patterns of disrespectful physician behavior at an academic medical center: implications for training, prevention, and remediation. Acad Med. 2018; 93: 1679–1685. 29319539 10.1097/ACM.0000000000002126

[12] Dabekaussen KF , Scheepers RA , Heineman E , et al. Health care professionals’ perceptions of unprofessional behaviour in the clinical workplace. PLoS One 2023; 18: e0280444. 36656827 10.1371/journal.pone.0280444 PMC9851503

[13] Dang D , Bae SH , Karlowicz KA , et al. Do clinician disruptive behaviors make an unsafe environment for patients? J Nurs Care Qual. 2016; 31: 115–123. 26323048 10.1097/NCQ.0000000000000150

[14] Geiderman JM , Moskop JC , Marco CA , et al. Civility in health care: a moral imperative. HEC Forum. 2022; 36: 245–257. 36547791 10.1007/s10730-022-09501-y PMC11070391

[15] Ulreich S , Harris RD , Sze G , et al. The disruptive radiologist. J Am Coll Radiol. 2015; 12: 800–804. 25920582 10.1016/j.jacr.2015.02.013

[16] Rosenstein AH and O'Daniel M . Impact and implications of disruptive behavior in the perioperative area. J Am Coll Surg. 2006; 203: 96–105. 16798492 10.1016/j.jamcollsurg.2006.03.027

[17] Bochatay N , Bajwa NM , Cullati S , et al. A multilevel analysis of professional conflicts in health care teams: insight for future training. Acad Med. 2017; 92: S84‐S92. 29065028 10.1097/ACM.0000000000001912

[18] Bae SH , Dang D , Karlowicz, et l . Triggers contributing to health care clinicians’ disruptive behaviors. J Patient Saf. 2020; 16: e148‐e155. 27811590 10.1097/PTS.0000000000000288

[19] Pavithra A , Sunderland N , Callen J , et al. Unprofessional behaviours experienced by hospital staff: qualitative analysis of narrative comments in a longitudinal survey across seven hospitals in Australia. BMC Health Serv Res. 2022; 22: 410. 35351097 10.1186/s12913-022-07763-3 PMC8962235

[20] Martinez W , Pichert JW , Hickson GB , et al. Qualitative content analysis of coworkers’ safety reports of unprofessional behavior by physicians and advanced practice professionals. J Patient Saf. 2021; 17: e883‐e889. 29547475 10.1097/PTS.0000000000000481

[21] Behaviors that undermine a culture of safety. Joint Commission Sentinel Event Alert . 2021. https://www.jointcommission.org/‐/media/tjc/documents/resources/patient‐safety‐topics/sentinel‐event/sea‐40‐intimidating‐disruptive‐behaviors‐final2.pdf 18686330

[22] MacDonald O . Disruptive Physician Behavior. American College of Physician Executives: QuantiaMD; 2011. https://www.kff.org/wp‐content/uploads/sites/2/2013/03/quantiamd_whitepaper_acpe_15may2011.pdf

[23] Zimmerman T and Amori G . J Healthc Risk Manag. 2011; 30: 5–6. 10.1002/jhrm.20055 21351191

[24] Harris JA and Byhoff E . Variations by state in physician disciplinary actions by US medical licensure boards. BMJ Qual Saf. 2017; 26: 200–208. 10.1136/bmjqs-2015-004974 27009311

[25] Cullen MJ , Konia MR , Borman‐Shopa EC , et al. Not all unprofessional behaviors are equal: the creation of a checklist of bad behaviors. Med Teach. 2017; 39: 85–91. 27670731 10.1080/0142159X.2016.1231917

[26] Reynolds NT . Disruptive physician behavior: use and misuse of the label. J Med Regul. 2012; 98: 8–19.

[27] Webb LE , Dmochowski RR , Moore IN , et al. Using coworker observations to promote accountability for disrespectful and unsafe behaviors by physicians and advanced practice professionals. J Comm J Qual Patient Saf. 2016; 42: 149–161. 10.1016/s1553-7250(16)42019-2 27025575

[28] Swiggart WH , Bills JL , Penberthy JK , Dewey CM , Worley LLM . A professional development course improves unprofessional physician behavior. J Comm J Qual Patient Saf. 2020;46:64–71. 10.1016/j.jcjq.2019.11.004 31899153

[29] Penberthy JK , Chhabra D , Ducar DM , et al. Impact of coping and communication skills program on physician burnout, quality of life, and emotional flooding. Saf Health Work. 2018; 9: 381–387. 30559985 10.1016/j.shaw.2018.02.005 PMC6284159

[30] Peisah C , Williams B , Hockey P , et al. Pragmatic systemic solutions to the wicked and persistent problem of the unprofessional disruptive physician in the health system. Healthcare. 2023; 11: 2455. 37685490 10.3390/healthcare11172455 PMC10487014

[31] Ramirez RN , Mayerson JL , Lewis VO , et al. AOA critical issues symposium: the disruptive physician: bad apple or toxic tree? J Bone Joint Surg Am. 2025; 107: 104–110. 39356743 10.2106/JBJS.23.01262

[32] Rosenstein AH . Physician disruptive behaviors: five year progress report. World J Clin Cases. 2015; 3: 930–934. 26601095 10.12998/wjcc.v3.i11.930 PMC4644894

[33] Williams BW and Williams MV . Understanding and remediating lapses in professionalism: lessons from the island of last resort. Ann Thorac Surg. 2020; 109: 317–324. 31479640 10.1016/j.athoracsur.2019.07.036

[34] Finlayson AJ , Dietrich MS , Neufeld R , et al. Restoring professionalism: the physician fitness‐for‐duty evaluation. Gen Hosp Psychiatry. 2013; 35: 659–663. 23910216 10.1016/j.genhosppsych.2013.06.009 PMC3923266

[35] Hastie MJ , Jalbout T , Ott Q , et al. Disruptive behavior in medicine: sources, impact, and management. Anesth Analg. 2020; 141: 1943–1949. 10.1213/ANE.0000000000005218 33009135

[36] Junga Z , Tritsch A , Singla M . How to “DEAL” with disruptive physician behavior. Gastroenterology. 2019; 157: 1469–1472. 31655032 10.1053/j.gastro.2019.10.021

[37] Lim S , Goh EY , Tay E , et al. Disruptive behavior in a high‐power distance culture and a three‐dimensional framework for curbing it. Health Care Manage Rev. 2022; 47: 133–143. 34009832 10.1097/HMR.0000000000000315 PMC8876433

[38] Veltman L . The disruptive physician: the risk manager's role. J Healthc Risk Manag. 1995;15(2):11‐16 10.1002/jhrm.5600150205 10155807

[39] Bernabeo EC , Chesluk B , Lynn L . Tiny moments matter: Promoting professionalism in everyday practice. J Contin Educ Health Prof. 2018; 38: 110–116. 29782368 10.1097/CEH.0000000000000202

[40] McCullough LB , Coverdale J , Chervenak FA . Professional virtue of civility and the responsibilities of medical educators and academic leaders. J Med Ethics. 2023; 49: 674–678. 36889908 10.1136/jme-2022-108735 PMC10579492

[41] Barnhoorn PC , Houtlosser M , Ottenhoff‐de Jonge MW , et al. A practical framework for remediating unprofessional behavior and for developing professionalism competencies and a professional identity. Med Teac. 2019; 41: 3. 10.1080/0142159X.2018.1464133 29703096

[42] Institute for Safe Medication Practices . 13 Ways to Address Disrespectful Behaviors in Healthcare: ISMP. . Accessed: April 1, 2024. https://home.ecri.org/blogs/ismp‐news/13‐ways‐to‐address‐disrespectful‐behaviors‐in‐healthcare‐ismp?_pos=3&_sid=0dafd2f9b&_ss=r

